# Association of ctDNA detection and recurrence assessment in patients with neoadjuvant treatment

**DOI:** 10.1002/cam4.6544

**Published:** 2023-09-25

**Authors:** Jiaxin Zhou, Haocong Mo, Dahai Hu, Xiaoxu Zhao, Hong Zhou, Jinghua Pan

**Affiliations:** ^1^ General Surgery The First Affiliated Hospital of Jinan University Guangzhou China; ^2^ International School Jinan University Guangzhou China; ^3^ Department of Physiology, School of Medicine Jinan University Guangzhou China; ^4^ Department of Gastrointestinal Surgery The Fifth Affiliated Hospital of Jinan University Heyuan China; ^5^ Department of Obstetrics and Gynecology The First Affiliated Hospital of Jinan University Guangzhou China

**Keywords:** biomarker, cancer, circulating tumor DNA, meta‐analysis, neoadjuvant treatment

## Abstract

**Background:**

The utilization of neoadjuvant therapy is progressively expanding in various clinical settings. However, the absence of a clinically validated biomarker to evaluate the treatment response remains a significant challenge in the field. Circulating tumor DNA (ctDNA) detection, a novel and emerging monitoring approach in the field of oncology, holds promise as a potential prognostic biomarker for patients with cancer. This meta‐analysis investigated the clinical significance of ctDNA detection as a predictive tool for cancer recurrence in patients receiving neoadjuvant treatment.

**Methods:**

A comprehensive systematic literature search was conducted using public databases to identify relevant studies that investigated the association between ctDNA detection and cancer recurrence in patients receiving neoadjuvant treatment. Hazard ratios (HRs) and their corresponding 95% confidence intervals (95% CI) were calculated to assess the relationship between cancer recurrence and relevant factors. Cancer recurrence was considered the primary outcome.

**Results:**

A total of 23 studies encompassing 1590 patients across eight different cancer types were included in the final analysis. Positive ctDNA detection was significantly associated with higher cancer recurrence, especially at post‐neoadjuvant treatment and post‐surgery time points. The risk values for the different cancer categories and geographic areas also differed significantly.

**Conclusion:**

Our comprehensive meta‐analysis revealed a significant positive correlation between ctDNA detection and a higher risk of cancer recurrence in patients receiving neoadjuvant treatment. In addition, the risk of recurrence was influenced by variations in cancer type, timing of detection, and geographic region. These findings highlight the promising clinical applicability of ctDNA as a prognostic marker and monitoring approach for patients with cancer. However, the precise mechanism is unknown and more evidence is needed for further research.

## 1 INTRODUCTION

Currently, cancer, which is one of the foremost causes of human mortality, exhibits a significantly high worldwide prevalence. The year 2020 witnessed a substantial escalation in the global burden of cancer, with over 19 million cases and approximately 9 million fatalities.^1^ These estimations underscore the urgent need for continued research and interventions to combat this devastating disease. Despite advancements in surgical techniques, chemotherapy, radiotherapy, and emerging modalities, which have undoubtedly enhanced survival rates and quality of life among patients with cancer, the persistently high incidence of local recurrence and distant metastasis necessitates further investigation and innovative therapeutic strategies to address this ongoing challenge. Therefore, there is an urgent demand for the development of novel biomarkers that can facilitate the detection of cancer recurrence and monitoring of treatment response, preferably through noninvasive and patient‐friendly approaches, to address the unmet clinical needs in this critical area.

Circulating tumor DNA (ctDNA) holds immense promise as a valuable tool for cancer management and detection, assessing treatment response, and potentially enabling early cancer detection. Circulating cell‐free DNA (ccfDNA), a constituent naturally present in blood at typically low concentrations, exhibits elevated levels in various circumstances such as exercise, trauma, and cancer.^2^, ^3^ ctDNA carries specific mutations, which can be isolated and detected in a wide range of bodily fluids, constituting a subset of ccfDNA with potential diagnostic and prognostic implications.^4^ It has shown great promise for detecting minimal residual disease (MRD) and predicting responses to specific therapies in clinical settings, while being minimally invasive and highly sensitive.^5^

Neoadjuvant treatment has gained significant prominence in cancer treatment, which is mainly employed prior to surgery to effectively decrease the occurrence of metastasis and tumor volume.^6^ Neoadjuvant treatment exhibits superior benefits across multiple dimensions for patients with cancer when compared to conventional adjuvant therapy. In the context of breast cancer research, noteworthy findings indicate that neoadjuvant treatment significantly contributes to tumor size reduction, increased rates of breast‐conserving surgery, and improved prognostic outcomes for patients with residual disease.^7^ Furthermore, neoadjuvant chemotherapy has demonstrated the ability to enhance treatment compliance, potentially leading to improved patient outcomes, while the occurrence of postoperative complications and treatment‐related toxicities may restrict adherence to adjuvant treatment.^8^ For instance, breast cancer mortality has emerged as the second most prominent cause of cancer‐related deaths in women.^9^ Neoadjuvant treatment represents a valuable therapeutic approach for patients with breast cancer, enabling higher rates of breast conservation and direct assessment of treatment efficacy.^10^

However, the existing literature on the correlation between ctDNA detection and cancer recurrence evaluation in patients undergoing neoadjuvant treatment is currently limited and subject to ongoing debate. In current clinical work, the assessment of pathological staging and microscopic residual disease score systems are the main approaches to estimate the risk of recurrence,^11^, ^12^, ^13^ which are limited and difficult to perform. Therefore, there is an urgent clinical need for novel biomarkers to identify the responses to neoadjuvant treatment and recurrence. In this meta‐analysis, we evaluated the potential role of ctDNA detection in predicting cancer recurrence.

## 2 MATERIALS AND METHODS

Our meta‐analysis was performed in accordance with the Preferred Reporting Items for Systematic Reviews and Meta‐Analyses guidelines. The International Prospective Register of Systematic Reviews registration number for this study is PROSPERO CRD 42023395312.

### 2.1 Data sources and literature searching

Five electronic databases, including PubMed, Cochrane Library, Embase, Web of Science, and JAMA, were symmetrically searched based on the MeSH words from the National Center of Biotechnology Information (NCBI). (((“Neoplasms”) AND “Circulating Tumor DNA”) AND “Neoadjuvant Treatment”) AND (“Recurrence” OR “Neoplasm Recurrence, Local”) were used as the search query. The patient, intervention, comparison, and outcome (PICO) framework was used, with no search restrictions. The search was performed independently by two authors, and any disagreements were resolved after discussion with a third author.

### 2.2 Inclusion and exclusion criteria

All of the authors formulated these criteria to identify eligible studies. The inclusion criteria were as follows: (I) publications on the recurrence of cancer patients treated with neoadjuvant treatment and ctDNA detection; (II) studies where participants were divided into two or more groups according to their ctDNA detection; (III) studies where participants underwent both neoadjuvant treatment and ctDNA detection; (IV) studies including sufficient and standard data; and (V) only English publications. The exclusion criteria were as follows: (I) duplicate publications and data; (II) literature with data from public databases; and (III) literature types such as reviews, case reports, meeting abstracts, and basic experimental research literature.

### 2.3 Data extraction and quality assessment

The screening of search results and data extraction were performed by two independent authors. Discrepancies were resolved through rigorous discussions. In cases of ongoing disputes, a third author was readily available for further resolution. All participants in the included studies underwent neoadjuvant treatment and ctDNA detection. The following information was collected in a predefined table, from each of the eligible studies, including author's name, publication year, country or area, the number of patients, study design, cancer type, neoadjuvant treatment program, ctDNA analysis method, ctDNA detection time points, ctDNA results, follow‐up duration, and recurrence outcomes. The authors were contacted in instances of data unavailability to retrieve the required information. The Newcastle–Ottawa scale (NOS) was used for the quality assessment of the included studies.

### 2.4 Outcomes and data analysis

The recurrence risk was the main outcome of our study. Recurrence details were collected from both the ctDNA‐positive and ctDNA‐negative participants. Review Manager 5.4 software for Mac was used to calculate the pooled hazard ratios (HRs) with corresponding 95% confidential intervals (CIs). To minimize the effects of heterogeneity, a random‐effects model was used to perform the dichotomous variance method. Statistical heterogeneity was assessed using the χ^2^ test and the *I* ^2^ test. Publication bias was estimated using a funnel plot, and sensitivity analysis was performed by removing literature from relatively low‐quality resources. *p* < 0.05 was considered statistically significant in this study. Moreover, to further explore the possible factors affecting this association, subgroup analyses including various cancer types, detection time points, and regions were performed.

## 3 RESULTS

### 3.1 Study identification

**FIGURE 1:**
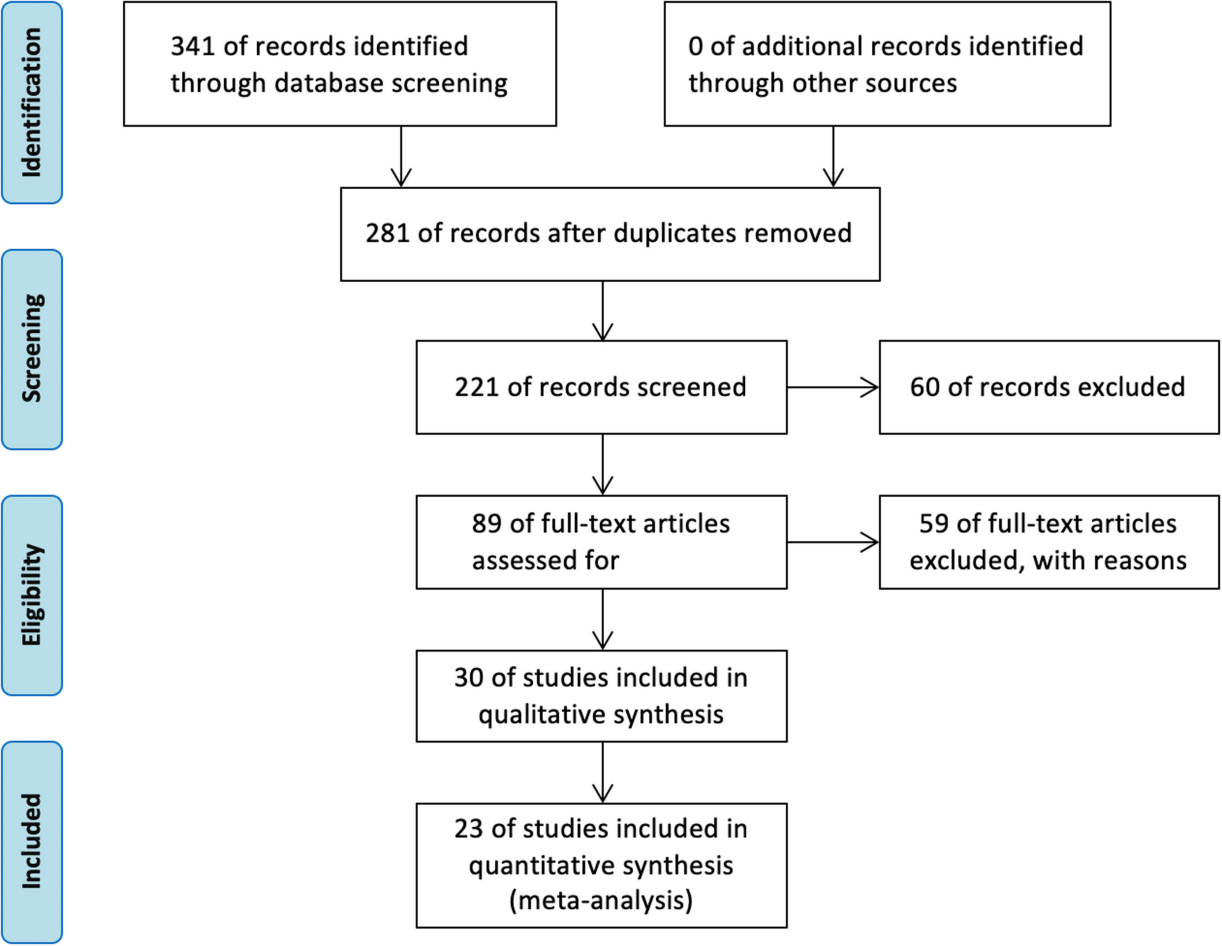
Flowchart of the literature search and selection.

The initial systemic literature search included 341 articles, comprising 61 articles from PubMed, 22 from the Cochrane Library, 171 from Embase, 72 from the Web of Science, and 15 from JAMA. After removing duplicates and studies that did not meet the requirements, 23 articles were selected (Figure 1).

### 3.2 Baseline characteristics of included research

The characteristics and details of the 23 included studies are summarized in Table 1. A total of 1590 patients, 8 cancer types (breast cancer, non‐small cell lung cancer, gastric cancer, colorectal cancer, renal cancer, esophageal cancer, melanoma, and bladder cancer), and 10 countries from 4 regions (America, Asia, Europe, and Australia) were included in these 23 studies, with publication year from 2016 to 2022. Among all the studies, 11 were prospective observational cohorts, 8 were retrospective cohorts, 2 were randomized controlled studies, and 2 were randomized controlled trials (RCTs). Of the studies included in the analysis, 11, 13, and 15 included pre‐neoadjuvant ctDNA detection (before neoadjuvant treatment), post‐neoadjuvant ctDNA detection (after neoadjuvant treatment but before surgery), and post‐surgery ctDNA detection (after surgery), respectively. The follow‐up duration was collected in 22 studies, ranging from 6.83 months to 61.2 months.

**TABLE 1:** Baseline characteristics of included studies.

Study	Year	Country	Follow‐up	Cancer type	NOS score
Alessandro Leal^14^	2020	America	Median: 42 months	Gastric cancer	8
Bernadett Szabados^15^	2022	England	Median: 25 months	Urothelial bladder cancer	5
Dengbo Ji^16^	2020	China	NA	Rectal cancer	8
Dongsheng Yue^17^	2020	China	Median: 6.83 months	Non‐small cell lung cancer	8
E. La Rocca^18^	2020	Italy	Median: 61.2 months	Breast cancer	5
E. Ortolan^19^	2022	Italy	Median: 36 months	Breast cancer	7
Emil Christensen^20^	2022	Denmark	Median: 96 months	Urothelial bladder cancer	9
Filipa Lynce^21^	2019	America	Median: 17 months	Breast cancer	5
Fre ´de ´ric Cailleux^22^	2019	America	Median: 36.36 months	Breast cancer	6
Georgina V Long^23^	2022	Australia	Median: 27 months	Melanoma	7
Haeyoung Kim^24^	2022	Korea	Median: 22 months	Breast cancer	5
Hiroyo Takahashi^25^	2019	Japan	Median: 23 months	Breast cancer	6
Jeanne Tie^26^	2021	Australia	Median: 50.5 months	Colorectal cancer	6
Jennifer Wo^27^	2016	America	Meidian: 23.1 months	Gastroesophageal cancer	7
Joana Vidal^28^	2021	Spain	Median: 38 months	Rectal cancer	7
Lisa S.M. Hofste^29^	2020	Netherlands	Median: 28 months	Rectal cancer	9
M.J.M. Magbanua^30^	2021	America	Median: 57.6 months	Breast cancer	7
Po‐Han Lin^31^	2022	America	Median: 61.2 months	Breast cancer	8
Shelize Khakoo^32^	2020	America	Median: 26.4 months	Rectal Cancer	5
Susan G. R. McDuff^33^	2021	America	Median: 20 months	Rectal cancer	7
V. F. Bonazzi^34^	2020	Australia	Median: 37 months	Esophageal cancer	7
Wenyang Liu^35^	2020	China	Median: 33.25 months	Rectal cancer	8
Yaqi Wang^36^	2022	China	Median: 21.5 months	Rectal cancer	8

Study	Number of patients	NAT treatment	Method of ctDNA analysis	ctDNA detection time points	Study type
Alessandro Leal^14^	50	Epirubicin, cisplatin, oxaliplatin, capecitabine	NGS, PCR	Post‐NAT	Randomized controlled study
Bernadett Szabados^15^	36	Cisplatin, atezolizumab	Multiplex PCR; NGS	Post‐surgery	Prospective study
Dengbo Ji^16^	46	Capecitabine alone, mFOLFOX6, CapeOx	NGS	Pre‐NAT	Retrospective study
	37			Post‐NAT	
	32			Post‐surgery	
Dongsheng Yue^17^	22	Nivolumab + platinum double chemotherapy 54%; docetaxel + cisplatin 18%; Pemetrexed + cisplatin 10%; nivolumab + ipilimumab 18%	ddPCR; UDS; LC‐MRD‐panel; NGS	Pre‐NAT	Retrospective study
	22			Post‐NAT	
	22			Post‐surgery	
E. La Rocca^18^	25	Anthracycline + taxane‐based (81.8%); Platinum‐based (12.1%) Anthracycline‐based (6.1%)	NGS; ddPCR	Post‐surgery	Retrospective study
E. Ortolan^19^	26	Anthracycline/taxane (80.6%) Anthracycline/taxane + platins (12.9%) Other (6.5%)	Primary‐tumor‐targeted gene sequencing; Patient‐ specific point mutations; ddPCR	Post‐NAT	Prospective study
	24			Post‐surgery	
Emil Christensen^20^	59	Gemcitabine + cisplatin (94%) Gemcitabine + carboplatin (1.5%) Gemcitabine (3%) Carboplatin+etoposide (1.5%)	WES; UDS; NGS	Pre‐NAT	Prospective study
	63			Post‐NAT	
	64			Post‐surgery	
Filipa Lynce^21^	30	Taxane + anthracyclines; Taxanes only	NA	Pre‐NAT	Randomized controlled study
Fre ´de ´ric Cailleux^22^	38	NA	WES; Bespoke multiplex PCR; NGS	Pre‐NAT	Retrospective
	42			Post‐NAT	
	40			Post‐surgery	
Georgina V Long^23^	35	dabrafenib + trametinib	NA	Pre‐NAT	RCT
Haeyoung Kim^24^	11	Capecitabine	Gene sequencing	Pre‐NAT	Prospective study
Hiroyo Takahashi^25^	54	Paclitaxel	OS‐MSP	Post‐surgery	Prospective study
Jeanne Tie^26^	54	Oxaliplatin‐based	Safe‐SeqS assay; Tumor tissue mutation analysis; Plasma sample mutation analysis	Pre‐NAT	Prospective study
	49			Post‐surgery	
Jennifer Wo^27^	16	FOLFIRINOX	Paired‐end sequencing; NGS	Post‐NAT	Prospective study
	14			Post‐surgery	
Joana Vidal^28^	52	Aflibercept+mFOLFOX6 (64%) mFOLFOX6 (36%)	NGS	Pre‐NAT	RCT
	45			Post‐NAT	
Lisa S.M. Hofste^29^	16	Carboplatin and paclitaxel + Gy radiation	UDS	Post‐surgery	Prospective study
M. J. M. Magbanua^30^	75	NA	UDS	Pre‐NAT	Retrospective study
	74			Post‐NAT	
Po‐Han Lin^31^	95	Anthracycline; taxane; anti‐Her2 target therapy	NGS	Post‐surgery	Retrospective study
Shelize Khakoo^32^	47	NA	dd PCR	Post‐NAT	Prospective study
Susan G. R. McDuff^33^	24	Capecitabine or infusion fluorouracil	NGS; dd PCR	Post‐NAT	Retrospective study
	19			Post‐surgery	
V. F. Bonazzi^34^	36	NA	Multiple tumor biopsies sequencing	Post‐surgery	Retrospective study
Wenyang Liu^35^	42	Capecitabine + oxaliplatin regimen	Multiplex PCR; LDS	Pre‐NAT	Prospective study
	35			During‐NAT	
	60			Post‐NAT	
Yaqi Wang^36^	103	Capecitabine + irinotecan	Panel sequencing	Post‐NAT	Prospective study
	103			Post‐surgery	

Abbreviations: ddPCR, droplet digital polymerase chain reaction; LC‐MRD, lung cancer‐specific minimal residual disease; LDS, low‐depth sequencing; NA, not available; NAT, neoadjuvant treatment; NGS, next‐generation sequencing; OS‐MSP, one‐step methylation‐specific polymerase chain reaction; PCR, polymerase chain reaction; RCT, randomized clinical trial; Safe‐SeqS, safe‐Sequencing; UDS, ultradeep sequencing; WES, whole‐exome sequencing.

#### 3.2.1 Positive‐ctDNA detection was positively correlated with higher recurrence possibility

**FIGURE 2:**
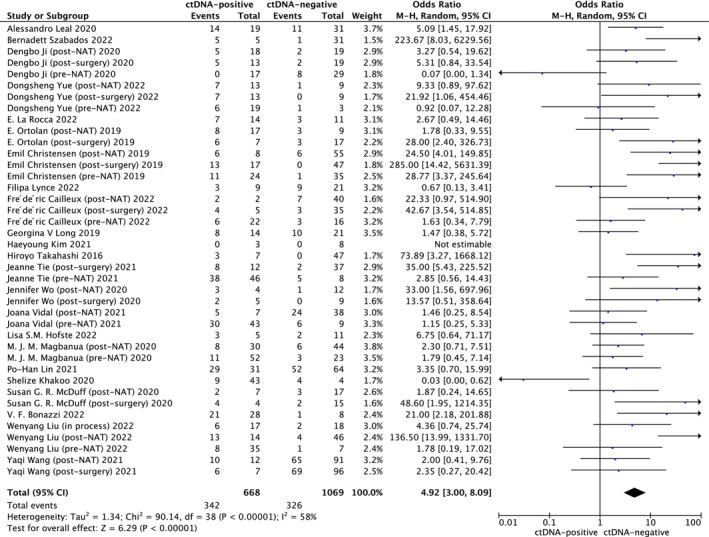
Forest plot showing the association between ctDNA detection and recurrence in cancer patients treated with neoadjuvant treatment. CI: confidential interval; Random: random‐effects model.

In the current study, a comprehensive analysis was conducted using all available data to investigate the association between ctDNAs and recurrence. The results revealed that the positive ctDNA detection result was strongly associated with a higher recurrence rate (HR: 4.92, 95% CI: 3.00–8.09, *p* < 0.001, *I* ^2^ = 58%; Figure 2).

### 3.3 The risk of recurrence varied across different cancer types

**FIGURE 3:**
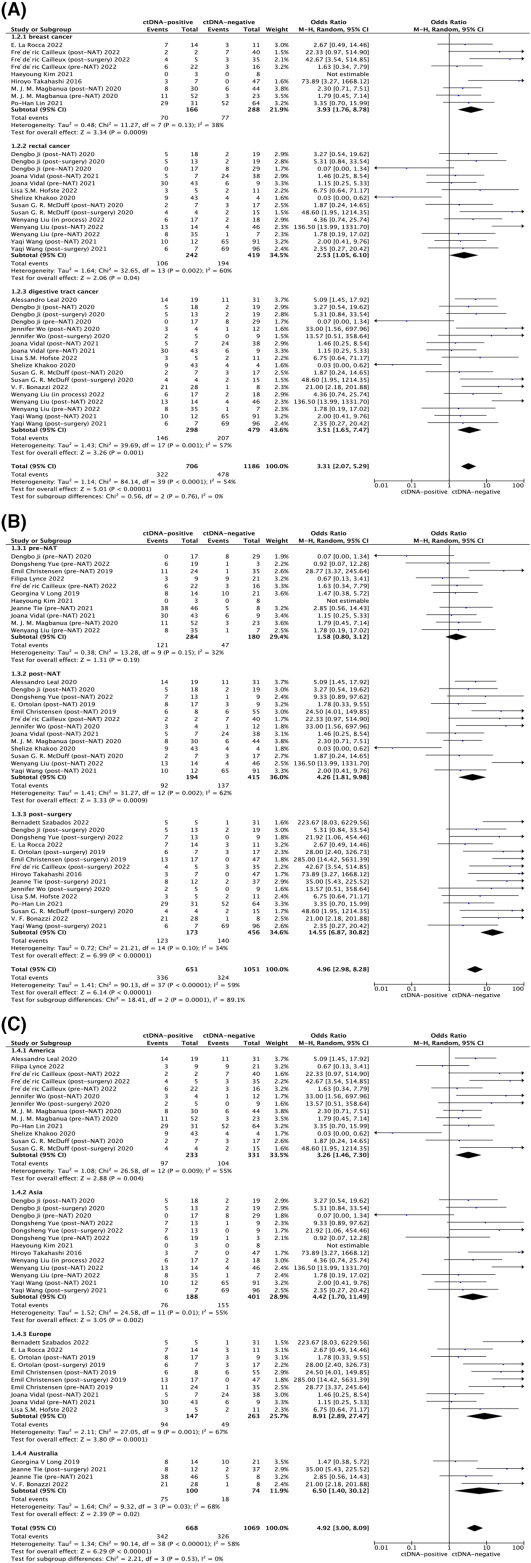
Subgroup analysis between ctDNA detection and recurrence in cancer patients treated with neoadjuvant treatment. (A) Subgroup analysis of cancer type. (B) Subgroup analysis of ctDNA detection time point. (C) Subgroup analysis of various area. CI, confidential interval; GI tract, gastrointestinal tract; NAT, neoadjuvant treatment; Random, random‐effects model.

Subgroup analyses were conducted to explore the association between ctDNA and recurrence across different cancer types, time points, and geographic regions, allowing for comprehensive evaluation of the data. (Table 2) In cancer category analysis, a statistical significance was observed in all the types including breast cancer (HR: 3.93, 95% CI: 1.76–8.78, *p* < 0.001, *I* ^2^ = 38%), rectal cancer (HR: 2.53, 95% CI: 1.05–6.10, *p* = 0.04, *I* ^2^ = 60%), and digestive tract cancer (HR: 3.51, 95% CI: 1.65–7.47, *p* = 0.001, *I* ^2^ = 57%). While all cancer types were positively related to the possibility of relapse, the HR values differed significantly. (Figure 3A).

**TABLE 2:** Subgroup analysis details.

Subgroup	(OR, 95% CI)	Number of studies	Number of patients	*p*	Heterogeneity				
					Tau^2^	Chi^2^	df	*I* ^2^ (%)	*p*
Cancer type									
Breast cancer	3.93 (1.76, 8.78)	9	454	<0.001	0.48	11.27	17	57	0.001
Rectal cancer	2.53 (1.05, 6.10)	14	661	0.04	1.64	32.65	13	60	0.002
Digestive tract cancer	3.51 (1.65, 7.57)	18	777	0.001	1.43	39.69	3	0	0.56
Time points									
Pre‐NAT	1.58 (0.80, 3.12)	11	464	0.19	0.38	13.28	9	32	0.15
Post‐NAT	4.26 (1.81, 9.98)	13	609	<0.001	1.41	31.27	12	62	0.002
Post‐surgery	14.55 (6.87, 30.82)	15	629	<0.001	0.72	21.21	14	34	0.10
Area									
America	3.26 (1.46, 7.30)	13	564	0.004	1.08	26.58	12	55	0.009
Asia	4.42 (1.70, 11.49)	13	589	0.002	1.52	24.58	11	55	0.01
Europe	8.91 (2.89, 27.47)	10	410	<0.001	2.11	27.05	9	67	0.001
Australia	6.50 (1.40, 30.12)	4	174	0.02	1.64	9.32	3	68	0.03

Abbreviations: CI, confidential interval; OR, odds ratio; NAT, neoadjuvant treatment.

### 3.4 Multi‐time points were related with recurrence rate

In the sub‐analysis between cancer recurrence and ctDNA detection time points presented in Figure 3B, a robust correlation was observed at the time points post‐neoadjuvant treatment (HR: 4.26, 95% CI: 1.81–9.98, *p* < 0.001, *I* ^2^ = 62%) and post‐surgery (HR: 14.55, 95% CI: 6.87–30.82, *p* < 0.001, *I* ^2^ = 34%), which indicated that a detectable ctDNA in patients who received the detection at post‐neoadjuvant treatment and post‐surgery time points was positively correlated with recurrence occurrence. However, no statistically significant differences were observed in pre‐neoadjuvant detection. (HR: 1.58, 95% CI: 0.80–3.12, *p* = 0.19, *I* ^2^ = 32%).

### 3.5 Relapse rate differed in various regions

Moreover, we conducted an analysis of diverse regions to explore potential variations in the association between ctDNA and cancer recurrence, which were also proved to be related with recurrence in America (HR: 3.26, 95% CI: 1.46–7.30, *p* = 0.004, *I* ^2^ = 55%), Asia (HR: 4.42, 95% CI: 1.70–11.49, *p* = 0.002, *I* ^2^ = 55%), Europe (HR: 8.91, 95% CI: 2.89–27.47, *p* < 0.001, *I* ^2^ = 67%), and Australia (HR: 6.50, 95% CI: 1.40–30.12, *p* = 0.02, *I* ^2^ = 68%) (Figure 3C).

### 3.6 Sensitivity analysis and publication bias

In the sensitivity analysis, the resulting trends remained the same as those in the above analysis after removing the five low‐quality comparisons, as shown in Figure S1.^15^, ^18^, ^21^, ^24^, ^32^ (HR: 5.42, 95% CI: 3.34–8.75, *p* < 0.001, *I* ^2^ = 51%) The NOS scores of 23 included literatures ranged from 5 to 9. Funnel plots were chosen to estimate publication bias in this study and no obvious bias was observed. (Figure S2).

## 4 DISCUSSION

Our meta‐analysis indicated that a positive ctDNA result in patients with cancer who were treated with neoadjuvant therapy is strongly correlated with a higher incidence of recurrence. Additionally, we observed substantial variations in the risk values for recurrence based on the different cancer types, ctDNA detection time points, and the patients' geographic regions. The results revealed that the cancer categories of breast and digestive tract cancer and four regional classifications (America, Asia, Europe, and Australia) were positively related to recurrence. Significant associations were observed only at the post‐neoadjuvant treatment and post‐surgery time points, with no statistically significant relationships identified in the pre‐neoadjuvant treatment period. To the best of our knowledge, this meta‐analysis is the most comprehensive study exploring the relationship between ctDNA detection in patients treated with neoadjuvant therapy and cancer recurrence. We included the maximum number of studies and subgroup analyses including cancer category, detection time points, and regions. In addition, to enhance credibility and reduce heterogeneity, we ensured that all participants had undergone both ctDNA detection and neoadjuvant treatment, thereby strengthening the validity of our findings.

ctDNA is a novel monitoring approach that works in MRD detection, treatment response prediction, and resistance mechanism evaluation.^37^, ^38^ Currently, neoadjuvant treatment is considered a promising therapy for cancer before surgery. The neoadjuvant approach provides an ideal opportunity to discover novel biomarkers that can accurately predict the response to the specific treatment administered.^39^, ^40^ In addition, it has been demonstrated to improve prognosis and quality of life in several cancer types, including breast cancer,^41^ locally advanced rectal cancer,^36^ and non‐small cell lung cancer.^42^ However, a valuable biomarker for assessing treatment efficiency is still needed. While several studies have explored ctDNA monitoring and its potential for recurrence prediction,^43^, ^44^ existing research specifically investigating its application in the context of patients receiving neoadjuvant treatment remains limited. Its predictive function for neoadjuvant treatment remains unknown and controversial. Shelize et al. found no difference in treatment response determined by RECIST between ctDNA‐positive and ctDNA‐negative patients at any time point.^45^ Similarly, Dengbo ji et al. observed no correlation between circulating free DNA (cfDNA) and cancer recurrence prediction.^16^ However, in Bernadett Szabados's research, ctDNA‐positive patients had a worse recurrence‐free survival than ctDNA‐negative patients.^15^ In our research, we conducted this meta‐analysis to confirm that positive ctDNA detection is strongly associated with a higher cancer recurrence rate in patients receiving neoadjuvant treatment. ctDNA is associated with a higher tumor proliferation index and a more aggressive subtype.^46^ Additionally, positive ctDNA detection indicated that tumors with high proliferation and increased cell turnover can release more tumor DNA fragments. Meanwhile, the detection of ctDNA in the bloodstream suggests the persistent presence of tumor cells or residual disease, thereby implying an elevated risk of cancer recurrence compared with cases where ctDNA is undetectable. This discovery indicates that ctDNA is a valuable biomarker for patients undergoing neoadjuvant treatment. Moreover, the status of ctDNA after neoadjuvant treatment can improve the performance of functional tumor volume as a predictor of metastatic recurrence.^22^ Neoadjuvant treatment reduces tumor size and tumor burden, while the concentration of ctDNA is positively correlated with tumor burden.^47^ Therefore, as neoadjuvant treatment leads to a decrease in tumor tissues, the concentration of ctDNA would also change correspondingly. Therefore, our study determined the key role of ctDNAs in monitoring the effects of neoadjuvant treatments.

The results also revealed that a positive ctDNA result was linked to various recurrence risks in diverse cancer types, which was reported for the first time. In patients with localized tumors, the ctDNA detection rate varies among the cancer types. ctDNAs were detected in 73% of patients with colorectal cancer, 57% of patients with gastroesophageal cancer, 48% of patients with pancreatic cancer, and 50% of patients with breast adenocarcinoma.^48^ Based on our comprehensive analysis, ctDNA demonstrates potential as a highly valuable biomarker for breast cancer patients with neoadjuvant treatment, as evidenced by the significantly elevated odds ratio (OR: 3.93) and risk ratio (RR: 2.66). Notably, in the context of digestive tract cancer, the presence of detectable ctDNA was also correlated with cancer recurrence, but with different recurrence risks (OR: 3.51, RR: 1.87). Qiu et al. observed that among preoperative ctDNA‐positive patients, those with adenocarcinoma had a shorter recurrence‐free survival, whereas this finding was not evident in squamous cell carcinoma.^38^ Therefore, ctDNA serves as a biomarker for assessing cancer prognosis and recurrence in patients with digestive tract malignancies; however, intrinsic mechanism research is still needed.

Current research on the efficacy of ctDNA detection as a biomarker at different time points remains controversial. Several studies have reported a meaningful relationship between ctDNA detection and recurrence prediction at pre‐neoadjuvant treatment,^14^, ^20^ post‐neoadjuvant treatment,^20^ and post‐surgery time points,^17^, ^20^ whereas Bernadett Szabados reported no recurrence in ctDNA‐positive patients at pre‐neoadjuvant and post‐neoadjuvant time points.^15^ After our analysis and investigations, the different risk values were observed at various time points. Interestingly, no statistical significance was observed when ctDNA detection was performed at the pre‐neoadjuvant treatment time point, while there was a strong association at the post‐neoadjuvant treatment and post‐surgery time points. Different stages of treatment and disease progression have an enhanced influence on the release and detection of ctDNA, consequently establishing a significant association. After neoadjuvant treatment or surgery, the tumor burden decreases but with the possibility of MRD. Thus, ctDNA analysis holds potential value in the neoadjuvant therapeutic context, as it allows for the identification of patients at a heightened risk of disease recurrence through the detection of MRD following neoadjuvant treatment.^49^ In addition, we performed a subgroup analysis of different regions. The results showed a significant association between all four areas and different HR values. This phenomenon can be attributed to multiple factors, including biological heterogeneity, diverse cancer therapy protocols, variability in treatment responses, and the influence of environmental factors across different geographic areas.

However, this study has several limitations. First, despite having the highest number of included studies compared to similar studies, the sample size may still be insufficient to provide a comprehensive analysis. Additionally, the range of cancer types included in this study may not be sufficiently comprehensive to fully capture the heterogeneity of ctDNA‐associated recurrence. In addition, different neoadjuvant treatment regimens may have contributed to the heterogeneity.

## 5 CONCLUSION

This meta‐analysis showed that positive ctDNA detection results in patients with cancer who received neoadjuvant treatment is positively correlated with an increased likelihood of recurrence. Highlighting ctDNA's potential as a recurrence predictor in clinical settings, especially following neoadjuvant treatment and surgery. Furthermore, our findings underscore the variable risk profiles across various cancer types and geographic regions. However, further research and clinical trials are imperative to explore the concrete mechanisms and the precise association between ctDNA detection and recurrence in patients with cancer undergoing neoadjuvant treatment.

## AUTHOR CONTRIBUTIONS

**Jiaxin Zhou:** Conceptualization (lead); data curation (lead); formal analysis (lead); investigation (lead); resources (lead); software (lead); writing – original draft (lead); writing – review and editing (lead). **Haocong Mo:** Formal analysis (equal); supervision (equal); validation (equal); writing – original draft (equal). **Dahai Hu:** Data curation (equal); formal analysis (equal); software (equal); writing – original draft (equal). **Xiaoxu Zhao:** Funding acquisition (equal); supervision (equal); validation (equal). **Hong Zhou:** Funding acquisition (equal); supervision (equal); validation (equal); writing – review and editing (equal). **Jinghua Pan:** Funding acquisition (equal); investigation (equal); methodology (equal); supervision (equal); writing – review and editing (equal).

## FUNDING INFORMATION

This work was partially supported by the Guangdong Province Medical Science and Technology Research Fund Project (A2021056), and the Guangdong Basic and Applied Basic Research Foundation (2020A1515110639).

## CONFLICT OF INTEREST STATEMENT

The authors declare that the research was conducted in the absence of any commercial or financial relationships that could be construed as a potential conflict of interest.

## Supporting information


**Figure S1** Forest plot of sensitivity analysis.
**Figure S2** (A) Funnel plot for the analysis between ctDNA detection and recurrence in cancer patients treated with neoadjuvant treatment. (B) Funnel plot for the subgroup analysis of different cancer categories between ctDNA detection and recurrence in cancer patients treated with neoadjuvant treatment. (C) Funnel plot for the subgroup analysis in various ctDNA detection time points between ctDNA detection and recurrence in cancer patients treated with neoadjuvant treatment. (D) Funnel plot for the subgroup analysis of different regions between ctDNA detection and recurrence in cancer patients treated with neoadjuvant treatment.

## Data Availability

The data and materials (including Supplementary Material) used to support the findings in this research are available from the corresponding author upon request.
