# Supplementary material for: Association of ctDNA detection and recurrence assessment in patients with neoadjuvant treatment
Source: Cancer Med. 2023 Sep 25;12(19):19794–806. doi: 10.1002/cam4.6544 (PMC10587978; doi:10.1002/cam4.6544)
Supplement: Supplementary file 1 — Figure S1 Forest plot of sensitivity analysis. Figure S2 (A) Funnel plot for the analysis between ctDNA detection and recurrence in cancer patients treated with neoadjuvant treatment. (B) Funnel plot for the subgroup analysis of different cancer categories between ctDNA detection and recurrence in cancer patients treated with neoadjuvant treatment. (C) Funnel plot for the subgroup analysis in various ctDNA detection time points between ctDNA detection and recurrence in cancer patients treated with neoadjuvant treatment. (D) Funnel plot for the subgroup analysis of different regions between ctDNA detection and recurrence in cancer patients treated with neoadjuvant treatment. [file CAM4-12-19794-s001.docx]

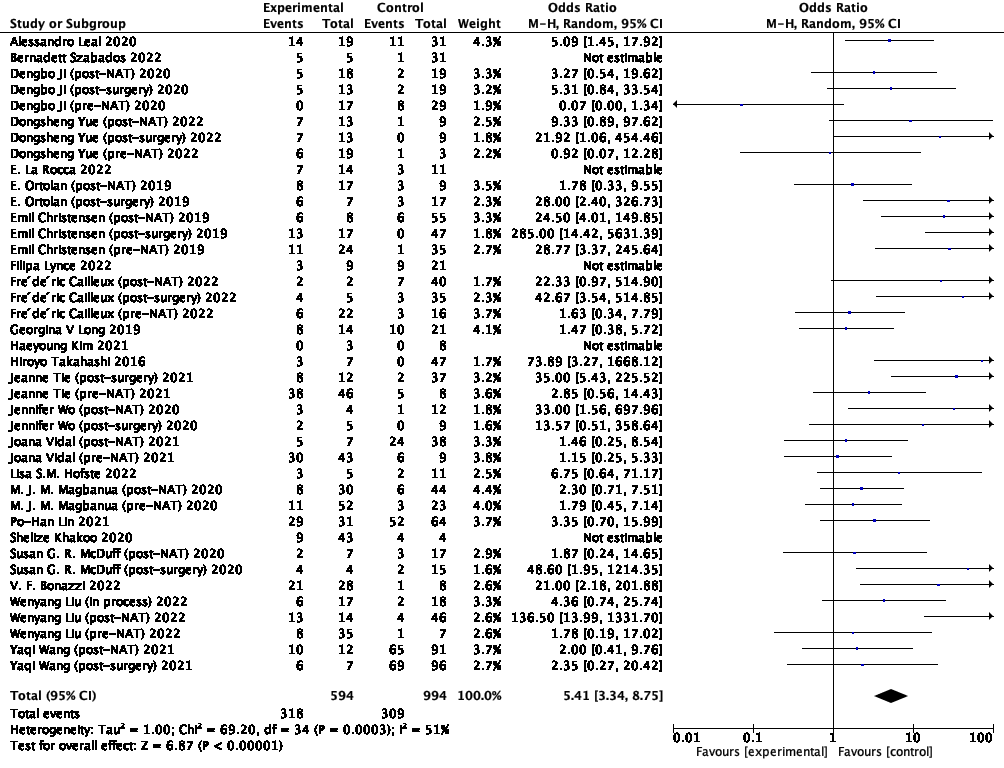


Supplementary Figure 1.

Forest plot of sensitivity analysis.


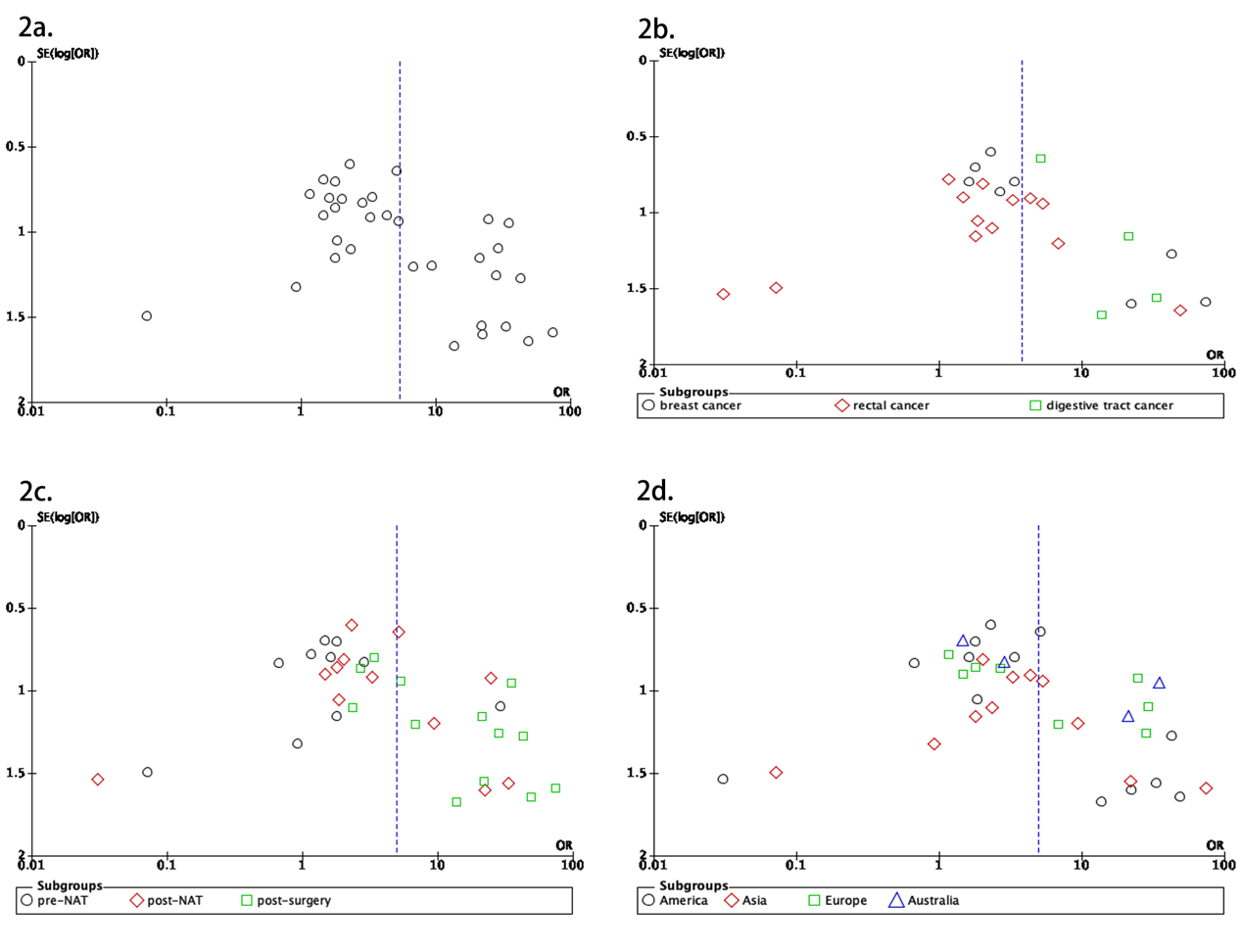


Supplementary Figure 2.

2a. Funnel plot for the analysis between ctDNA detection and recurrence in cancer patients treated with neoadjuvant treatment.

2b. Funnel plot for the subgroup analysis of different cancer categories between ctDNA detection and recurrence in cancer patients treated with neoadjuvant treatment.

2c. Funnel plot for the subgroup analysis in various ctDNA detection timepoints between ctDNA detection and recurrence in cancer patients treated with neoadjuvant treatment.

2d. Funnel plot for the subgroup analysis of different regions between ctDNA detection and recurrence in cancer patients treated with neoadjuvant treatment.
